# Evaluation of Hemodynamic Change by Indocyanine Green-FLOW 800 Videoangiography Mapping: Prediction of Hyperperfusion Syndrome in Patients with Moyamoya Disease

**DOI:** 10.1155/2020/8561609

**Published:** 2020-08-11

**Authors:** Xin Zhang, Wei Ni, Rui Feng, Yanjiang Li, Yu Lei, Ding Xia, Peng Gao, Shaoxuan Yang, Yuxiang Gu

**Affiliations:** ^1^Department of Neurosurgery, Huashan Hospital, Fudan University, Shanghai 200040, China; ^2^Department of Radiology, Huashan Hospital, Fudan University, Shanghai 200040, China

## Abstract

**Objective:**

Hyperperfusion syndrome (HPS) after bypass surgery for moyamoya disease (MMD) mainly results from redistribution of blood flow, which leads to poor outcomes, while effective methods to predict HPS are still lacking. Indocyanine green (ICG) videoangiography can assess regional cerebral blood flow changes semiquantitatively with the application of FLOW 800 software. The purpose of this study was to investigate whether the intraoperative evaluation of local hemodynamic changes around anastomotic sites using FLOW 800 videoangiography mapping can predict the incidence of HPS and clinical outcomes.

**Methods:**

Of the patients who were diagnosed with MMD in our hospital between August 2018 and December 2019, who underwent superficial temporal artery-middle cerebral artery bypass surgeries, we investigated 65 hemispheres (in 62 patients) in which intraoperative ICG analysis was performed using FLOW 800 (Zeiss Meditec, Oberkochen, Germany) to evaluate the local cerebral hemodynamics before and after anastomosis. Regions of interest were set at more than 2 points on the brain surface according to the location and situation of recipient arteries in the surgical area. Peak cerebral blood volume (CBV), regional cerebral blood flow (CBF), and time to peak (TTP) were calculated from the selected points. As the data were available intraoperatively, anastomoses were performed in a suitable area. According to the occurrence of HPS, patients were divided into the asymptomatic and symptomatic groups, from which hemodynamic parameters were compared. Furthermore, ROC analysis was performed to determine the diagnostic accuracy of change rates in CBV, CBF, and TTP (i.e., *Δ*CBV, *Δ*CBF, and *Δ*TTP) for predicting HPS.

**Results:**

Data from the 62 patients were analyzed, and all patients were closely assessed during hospitalization after the procedures. The values of *Δ*CBV and *Δ*CBF were significantly higher in the symptomatic group (*p* < 0.01), while *Δ*TTP is slightly lower in the symptomatic group with no statistical differences (*p* = 0.72). Hemodynamic parameters including *Δ*CBV and *Δ*CBF, calculated by FLOW 800, had high sensitivity and specificity according to the ROC curve (*Δ*CBV: AUC = 0.743, 95% CI, 0.605–0.881, *p* = 0.002; *Δ*CBF: AUC = 0.852, 95% CI, 0.750–0.954, *p* < 0.01), which could be used as predictors for HPS.

**Conclusions:**

Intraoperative ICG-FLOW 800 videoangiography mapping is a safe method which can reflect hemodynamic characteristics in the surgical area for MMD, the findings of which correlate with the occurrence of HPS. Parameters including *Δ*CBV and *Δ*CBF are proven to be efficient in the prediction of HPS.

## 1. Introduction

Moyamoya disease (MMD) is characterized by progressive stenosis to occlusion at the terminal of the internal carotid arteries associated with abnormal neovascularization, of which the cause remains unknown [1]. Surgical revascularization strategy, represented by a superficial temporal artery to middle cerebral artery (STA-MCA) anastomosis, improves cerebral blood supply and can decrease the risk of future infarction and hemorrhage [2, 3]. However, postoperative hyperperfusion syndrome (HPS), such as aphasia, epileptic seizures, and even new cerebral hemorrhage or ischemia, is experienced frequently in the acute phase after such surgeries [4]. With the evidence of neuroimaging, multiple literatures [5–7] have discussed local hemodynamic dysfunction as one of the main causes of such neurologic deficits. Besides, hypotheses such as watershed shift and inflammation have also been suggested. Although the physiological mechanisms behind HPS remain unclear, different varieties of clinical manifestation occur up to 50% of patients after bypass surgeries and progress to irreversible sequelae [4]. If the occurrence of HPS could be predicted, early intervention might be possible in order to reduce neurologic deficits. Accordingly, the elucidation of the predictors of HPS is desirable. Many studies [6, 8] have confirmed hemodynamic changes after bypass surgery, which seems to be a predictor of HPS. However, previous reports have only examined the relationship between the indirect parameters of hemodynamics and postoperative complications. Therefore, sufficient evidence and appropriate method are still lacking for evaluating the correlation between hemodynamic dysfunction and clinical outcomes.

FLOW 800 (Zeiss Meditec, Oberkochen, Germany) is specialized software for analyzing indocyanine green (ICG) videoangiography in a semiquantitative manner through color delay mapping and data analysis, which identifies the direction and different characteristics of blood flow via fluorescence dynamics. ICG-FLOW 800 represents a new modality that can set different regions of interest during surgery for arbitrary shapes and sites and then calculate dynamic factors [9]. In particular, the microvascular structure and fluorescence factors of the color map are available instantly during the procedure, which can provide direct parameters related to the local cerebral hemodynamics, such as cerebral blood flow (CBF), cerebral blood volume (CBV), and time to peak (TTP), and consequently guide immediate intraoperative decisions. In this study, we aimed to evaluate hemodynamic changes around anastomotic sites from before and after bypass surgery using the ICG-FLOW 800 videoangiography mapping method, while additionally confirming whether these changes could predict the incidence of HPS.

## 2. Methods

### 2.1. Patients

We retrospectively investigated 20 hemispheres in 19 patients who were diagnosed as having MMD and then underwent STA-MCA bypass surgery in our hospital from August 2018 to December 2019. All of these patients presented as different kinds of HPS symptoms after the operation and confirmed by cerebral perfusion imaging, among which there were no cases of cerebral hemorrhage or infarction on postoperative CT or MRI. Meanwhile, 45 hemispheres in 43 patients without HPS in the same period were included as a normal control in this study. Intraoperative ICG analysis was performed using FLOW 800 in all cases with high qualities of images. All of the patients met the Chinese guidelines for the diagnosis and treatment of MMD and moyamoya syndrome set by the Stroke Prevention Project Committee, National Health and Family Planning Commission, China. Each patient underwent cerebral perfusion imaging to confirm the hypoperfusion hemisphere and neurological test using the modified Rankin Scale (mRS) before the procedure. This study was approved by the Institutional Review Board of Huashan Hospital, Fudan University. Informed consent was obtained from the study participants.

### 2.2. Surgical Procedures and ICG-FLOW 800 Analysis

**Figure 1:**
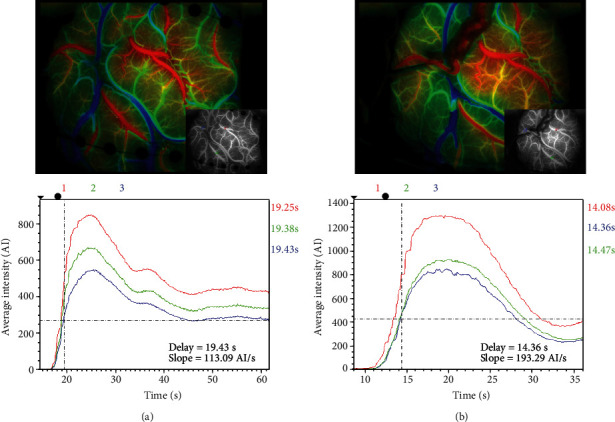
ICG was performed after a craniotomy to identify alternative recipient arteries in the surgical area (a). Color mapping was available to locate the hypoperfusion area for anastomosis. Regions of interest were set at the artery branch and parameters, such as cerebral blood flow (CBF, slope), cerebral blood volume (CBV, peak), and time to peak (TTP, delay time), which could be calculated and compared to confirm local cerebral hemodynamics for surgical decision making. Postoperative ICG-FLOW 800 was performed again (b) to confirm the patency of the anastomosis and to compare blood flow changes in the previously selected area.

All of the surgeries were performed by the same experienced neurosurgeon (Y.X.G.). ICG was performed after a frontotemporal craniotomy to identify the alternative recipient arteries corresponding to the diameter and length of the STA graft artery. Each case was confirmed with more than two optional recipient arteries in the surgical area. ICG was performed with 3 times magnification of a microscope to make sure the surgical area was covered. Color mapping was available with the application of FLOW 800 to evaluate hypoperfusion areas for anastomosis. As the regions of interest (ROIs) were set at the artery branches in these areas, several parameters, such as CBV, CBF, and TTP, could be calculated and compared to confirm local cerebral hemodynamics for surgical decision making. The STA graft was anastomosed to the MCA (M4 segment) in an end-to-side manner. Postoperative ICG-FLOW 800 was performed again to confirm the bypass patency and to make a comparison of the blood flow changes in the previously selected area (Figure 1).

### 2.3. Postoperative Evaluation and Management

After surgery, all patients were given fluid therapy and blood pressure monitoring to ensure enough circulation volume. Computed tomography (CT) and magnetic resonance imaging (MRI) were performed after the operations when the patients experienced new symptoms during the postoperative period. Postoperative complications and mRS score were recorded in detail. Both neuroimaging and neurological testing were compared for each patient within 2 weeks after the operation. A digital subtraction angiography (DSA) follow-up was performed about 6 months after the procedure.

### 2.4. Statistical Analysis

The change rate of parameters from the FLOW 800 was defined as

(1) $$\begin{array}{c} \Delta \text{Value}\left(\%\right)=\frac{\text{Postoperative Value}-\text{Preoperative Value}}{\text{Preoperative Value}}\times 100\%. \end{array}$$

According to the occurrence of HPS, patients were divided into the asymptomatic group and th symptomatic group. Demographics and hemodynamic parameters were compared between the two groups using the *χ*^2^ or unpaired *t*-test, as appropriate. Predictive specificity and sensitivity of each parameter were evaluated by receiver operating curve (ROC) analysis. Diagnostic accuracy was also determined by calculating the largest possible area under the curve (AUC) in ROC analysis. The optimal threshold value of hemodynamic parameters to predict HPS was determined by using Yoden's index analysis. *p* < 0.05 was considered statistically different in the univariate analyses. Database management and statistical analyses were performed via SPSS 21.0 software (SPSS Inc.).

## 3. Results

### 3.1. Demographics

In total, 36 male patients and 26 female patients (24 to 67 years old, average 47.16 years old, median 48 years old) were included in this study (Table 1). All the cases showed good patency of anastomosis from the DSA follow-up about 6 months after the procedure. For the symptomatic group, various kinds of HPS symptoms, such as limb numbness, aphasia, and epileptic seizures, occurred with a mRS score increase between 1 and 3 in 20 of the 65 hemispheres (30.8%) after revascularization. The asymptomatic group including the remaining 45 cases (69.2%) presented with either no symptoms, mild dizziness, or headaches without mRS score increase. With regard to age or the operated side, the two groups did not differ significantly (*p* > 0.1). Meanwhile, the present study showed that the rate of complication appeared higher in male patients than in female (*p* = 0.004).

### 3.2. Differences of Hemodynamic Parameters in Subgroups

**Figure 2:**
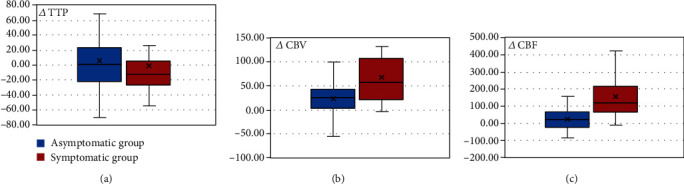
Subgroup analysis of the hemodynamic parameters (*Δ*CBF, *Δ*CBV, and *Δ*TTP) between the asymptomatic group (blue box) and the symptomatic group (red box). *Δ*TTP was slightly lower (-0.90% vs. 6.19%, *p* = 0.559) in the symptomatic group compared with the asymptomatic group, while *Δ*CBV (68.21% vs. 23.63%, *p* < 0.01) and *Δ*CBF (158.08% vs. 23.98%, *p* < 0.01) were significantly higher in the symptomatic group.

Each parameter was compared between the symptomatic and asymptomatic groups (Figure 2). In the symptomatic group, hemodynamic values were as follows: *Δ*TTP, −0.9% ± 48.94%; *Δ*CBV, 68.21% ± 58.02%; and *Δ*CBF, 158.08% ± 128.22%. In the asymptomatic group, hemodynamic values were as follows: *Δ*TTP, 6.19% ± 43.13%; *Δ*CBV, 23.63% ± 31.08%; and *Δ*CBF, 23.98% ± 56.42%. *Δ*TTP was slightly lower (*p* = 0.559) in the symptomatic group compared with the asymptomatic group. On the contrary, *Δ*CBV and *Δ*CBF were significantly higher (*p* < 0.01) in the symptomatic group.

### 3.3. ROC Analyses for Hemodynamic Parameters

**Figure 3:**
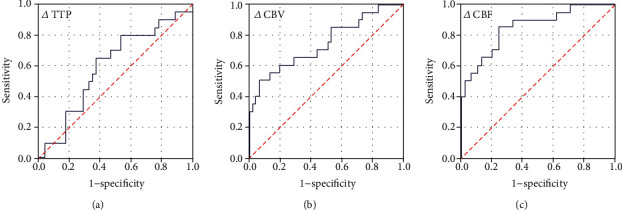
ROC analysis for each hemodynamic parameter correlated with clinical outcome. Results showed that diagnostic accuracy of *Δ*TTP was low (AUC = 0.597). As for *Δ*CBV, the AUC was 0.743 (95% CI, 0.605–0.881, *p* = 0.002), with the best threshold of 66.35 (sensitivity = 0.5, 1–specificity = 0.067, Yoden's index: 0.433). Similarly, the AUC was 0.852 for *Δ*CBF (95% CI, 0.750–0.954, *p* < 0.01), and the best threshold was 62.06 (sensitivity = 0.85, 1–specificity = 0.067, Yoden's index: 0.606).

Each parameter correlated with clinical manifestation was analyzed by using the ROC curve (Figure 3). For *Δ*CBV, the AUC was 0.743 (95% CI, 0.605–0.881, *p* = 0.002) and the best threshold was 66.35% to identify patients with complication (sensitivity: 50.0%, specificity: 93.3%, Yoden's index: 0.433). Similarly, the AUC was 0.852 for *Δ*CBF (95% CI, 0.750–0.954, *p* < 0.01), and the best threshold was 62.06% (sensitivity: 85.0%, specificity: 75.6%, Yoden's index: 0.606). While for *Δ*TTP, the AUC was 0.597 (95% CI, 0.449–0.745, *p* = 0.216), by which diagnostic accuracy was not enough.

### 3.4. Illustrative Cases

**Figure 4:**
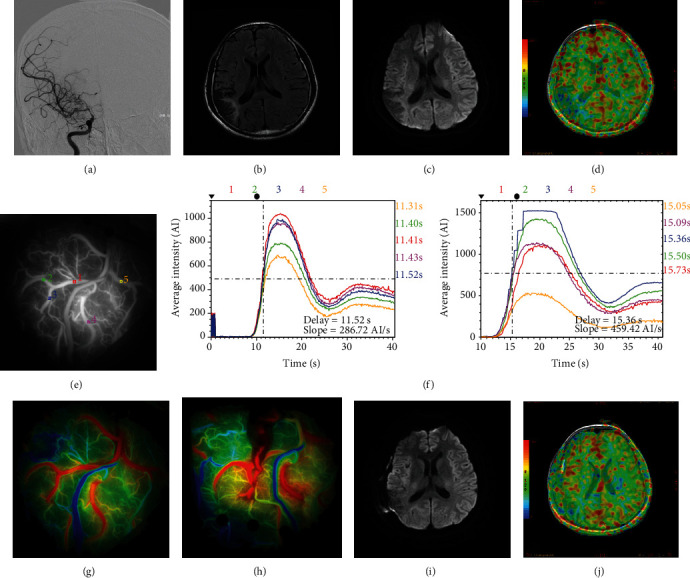
DSA showed occlusion of R-ACA and stenosis of R-MCA with moyamoya vessel formation (a). MR scan revealed old infarction on right temporal-occipital lobe (b, c). ASL confirmed hypoperfusion in the right hemisphere (d). Recipient artery was selected from five optional vessels from ICG-FLOW 800 mapping (e, g). Anastomosis was performed in vessel 3 (blue box), and hemodynamic analysis showed an obvious increase of *Δ*CBF and *Δ*CBV (f, h). Three days after the procedure, the patients suffered from an epileptic seizure and were controlled by benzodiazepine. MR follow-up confirmed HPS with the evidence of increasing blood flow but not new infarction (i, j).

A 31-year-old man was admitted to our hospital with a blurred vision. Preoperative MR scan revealed old infarction on the right temporal-occipital lobe. DSA demonstrated occlusion of the right anterior cerebral artery and stenosis of the right middle cerebral artery with compensatory vessel formation. Cerebral perfusion confirmed hypoperfusion in the right hemisphere. Right STA-MCA bypass combined with EDMS was performed, and the recipient artery was selected from five optional vessels according to the length and distribution of STA via ICG-FLOW 800 videoangiography mapping. Anastomosis was performed in vessel 3 (blue box), and hemodynamic analysis showed an obvious increase of *Δ*CBF and *Δ*CBV. Three days after the procedure, the patients suffered from an epileptic seizure and were controlled by benzodiazepine. An MR follow-up confirmed HPS with the evidence of increasing blood flow but not new infarction (Figure 4).

## 4. Discussion

Our analysis, performed using ICG-FLOW 800, showed that there were differences in the change rates of hemodynamic parameters (i.e., *Δ*CBF, *Δ*CBV, and *Δ*TTP) in patients with HPS compared with normal ones. Both *Δ*CBV and *Δ*CBF had high diagnostic accuracy, which could become optional predictive factors for the high-risk population with HPS. HPS was more likely to occur as *Δ*CBV became larger than 66.35% or *Δ*CBV became larger than 62.06%. More prospective studies need to be done to confirm this result.

Dysfunction of cerebral blood perfusion has been reported to occur when the local hemodynamic change caused by bypass surgery is excessively large and can result in postoperative neurological disorders and poor clinical outcomes [10]. Multiple literatures [6, 7] have reported that compensatory vessels were originally in a vasoparalytic state due to chronic ischemia, and excessive hemodynamic change acts as a stimulus that can cause HPS. From this, it could be inferred that neurologic deficits are more likely to occur as the local hemodynamic change becomes larger. The evaluation of hemodynamic changes in MMD from previous studies [11–13] has mainly focused on the hemisphere and regional cerebral perfusion changes by means of CT perfusion and multimodal MRI. These kinds of evaluations can assess the change of blood flow quantitively and predict the occurrence of HPS. Unfortunately, an efficient method with enough spatial and temporal resolution, which can be applied during the operation to predict HPS after a procedure, is still lacking [14]. Besides, choices for the recipient cortical artery in previous bypass surgeries have been generally based on the experience of the operator, along with a lack of theoretical foundation. In fact, postoperative HPS due to hemodynamic dysfunction, including hyperperfusion and watershed shift phenomenon, mainly result from a disturbance of the previous balance of blood flow [15]. It is important to find a solution for assisting surgical decision making and choosing an appropriate recipient artery.

FLOW 800 (Zeiss Meditec, Oberkochen, Germany) is a relatively new software tool integrated into the operative microscope that can be used to assess temporal distribution dynamics of ICG dye infusion to reflect the local blood flow index. Local hemodynamic characters can be evaluated intraoperatively, from which surgical decisions can be made for a suitable recipient artery with objective evidence. The maximum intensity map shows the maximum of fluorescence reached at each point of the image in a grayscale representation. These map representations summarily show how high the maximum fluorescence was during the observed period, which allowed the operator a good overview in a static mode of observation to see different blood volume conditions through fluorescence [16]. The spread of fluorescence in the areas observed was as expected, and the initial decision for the optional arteries could be made. After that, delay time mapping led to a representation of a pseudocolor image that provided a direct signal to show different blood flow arrival times. The early occurrence of the vessel initially showed in red and then later showed in blue. With this method, the operator could distinguish each optional recipient artery in the surgical area visually and make comparisons from the angle of the arrival time for each vessel. The operator could set the ROIs to adapt to the structure of these vessels and autocalculate the CBF, CBV, and TTP of every ROI diagram and provide related information on blood flow accordingly [17]. From this, the operator could evaluate the different hemodynamic parameters quantitively and choose the appropriate recipient artery. Different delay times revealed changes in cerebral blood flow and perfusion conditions intraoperatively before and after anastomosis.

This system automatically calculates the maximum fluorescent intensity (average intensity, also represented as CBV), time to maximum intensity (delay time, also represented as TTP), and slope of the intensity curve at this timepoint (slope, also represented as CBF) at the user-defined ROI within the operative field. Both color mapping and each semiquantitative hemodynamic parameter were available intraoperatively and could help surgeons to inform a surgical decision. We creatively apply this method in the bypass surgeries for MMD to localize the hypoperfusion area with objective hemodynamic parameters. As soon as an anastomosis is completed, the same protocol of ICG-FLOW 800 mapping can be performed to examine changes in perfusion at the surgical area in order to make comparisons for each parameter in the same ROIs. A previous study [16], using FLOW 800 for cerebral bypass, has demonstrated that various manually calculated and automated parameters can detect changes in pre- and postoperative perfusion and predict the risk of HPS. The reproducibility, and therefore clinical importance of these parameters, nonetheless remains unknown. From this study, we can forecast the occurrence of HPS after revascularization to a certain degree with the application of ICG-FLOW 800 mapping and prevent it in advance by means of increasing circulation volume, enhancing dehydration, and eliminating oxygen free radicals to improve clinical outcomes.

Notably, there are certain limitations to the present study. Primarily, ICG-FLOW 800 can only measure hemodynamic factors in the cortical region other than the vessel network, which may not be able to represent the global blood flow reconstruction sufficiently. Second, considering the small sample size of this study, participants were divided based on clinical symptom and correlated with ICG-FLOW 800 analyses. Some other factors including injection speed and position of ICG can also influence the value of each parameter. A certain anesthesiologist was arranged to standardized injection speed and position, and *Δ*value of each parameter can also remove interference from other factors. In fact, the changes of hemodynamics after bypass were complicated, which may be confirmed in further investigations with an increased cohort. As a preliminary study for modified surgical anastomosis series of researches, the application of ICG-FLOW 800 videoangiography mapping can evaluate and compare regional blood flow, which is an alternative model to reflect cerebral angioarchitecture and hemodynamics. Still, the distribution of global and regional perfusion, metabolism, and neuronal activity are also important influencing factors for surgical decision making in bypass surgeries. With the evidence of multimodal neuroimaging, the clinical outcome of bypass surgery may become much better with a lower complication rate.

In this work, we compared hemodynamic levels by means of ICG-FLOW 800 mapping in different groups and found a significant difference. Furthermore, we assessed the diagnostic accuracy for different hemodynamic parameters using ROC analysis and found efficient predictive factors as well as their threshold value for HPS. More comparative studies are needed to confirm these results.

## 5. Conclusion

ICG-FLOW 800 mapping can reflect hemodynamic characteristics in the surgical area for MMD intraoperatively, which is helpful for surgical decision making. Besides, changes in regional hemodynamic parameters correlate with the occurrence of HPS. Parameters including *Δ*CBV and *Δ*CBF are proven to be with high diagnostic accuracy and become optional predictive factors for the high-risk population with HPS.

## Figures and Tables

**Table 1:** Comparison of clinical parameters between groups with and without HPS.

Parameter	Perioperative complication		*p* value
	Yes	No	
Hemisphere	20	45	
Mean age (yrs)	46.55 ± 9.78	47.38 ± 11.34	0.778
Sex			
Male (%)	17 (85.0)	21 (46.7)	0.004^∗^
Female (%)	3 (15.0)	24 (53.3)	
Operated side			
Right (%)	8 (40.0)	22 (48.9)	0.507
Left (%)	12 (60.0)	23 (51.1)	

Values are numbers of patients or hemispheres unless otherwise indicated. Mean values are given with SDs.

## Data Availability

The data used to support the findings of this study are available from the corresponding author upon request.

## References

[1] Fujimura M., Bang O. Y., Kim J. S. (2016). Moyamoya disease. *Frontiers of Neurology and Neuroscience*.

[2] Funaki T., Takahashi J. C., Houkin K. (2020). Effect of choroidal collateral vessels on de novo hemorrhage in moyamoya disease: analysis of nonhemorrhagic hemispheres in the Japan Adult Moyamoya Trial. *Journal of Neurosurgery*.

[3] Lei Y., Li Y. J., Guo Q. H. (2017). Postoperative executive function in adult moyamoya disease: a preliminary study of its functional anatomy and behavioral correlates. *Journal of Neurosurgery*.

[4] Kim T., Oh C. W., Bang J. S., Kim J. E., Cho W. S. (2016). Moyamoya disease: treatment and outcomes. *Journal of Stroke*.

[5] Uda K., Araki Y., Muraoka S. (2018). Intraoperative evaluation of local cerebral hemodynamic change by indocyanine green videoangiography: prediction of incidence and duration of postoperative transient neurological events in patients with moyamoya disease. *Journal of Neurosurgery*.

[6] Yang T., Higashino Y., Kataoka H. (2018). Correlation between reduction in microvascular transit time after superficial temporal artery-middle cerebral artery bypass surgery for moyamoya disease and the development of postoperative hyperperfusion syndrome. *Journal of Neurosurgery*.

[7] Egashira Y., Yamauchi K., Enomoto Y., Nakayama N., Yoshimura S., Iwama T. (2017). Disruption of cortical arterial network is associated with the severity of transient neurologic events after direct bypass surgery in adult moyamoya disease. *World Neurosurgery*.

[8] Czabanka M., Peña-Tapia P., Schubert G. A. (2009). Clinical implications of cortical microvasculature in adult moyamoya disease. *Journal of Cerebral Blood Flow and Metabolism*.

[9] Shah K. J., Cohen-Gadol A. A. (2019). The application of FLOW 800 ICG videoangiography color maps for neurovascular surgery and intraoperative decision making. *World Neurosurgery*.

[10] Kazumata K., Uchino H., Tokairin K. (2018). Cerebral hyperperfusion syndrome after revascularization surgery in moyamoya disease: region-symptom mapping and estimating a critical threshold. *World Neurosurgery*.

[11] Wang X., Chong Z., Guo X. (2019). Evaluation of hemodynamics before and after revascularization in hemorrhagic moyamoya disease: a computed tomography perfusion imaging case study. *World Neurosurgery*.

[12] Togao O., Hiwatashi A., Obara M. (2018). 4D ASL-based MR angiography for visualization of distal arteries and leptomeningeal collateral vessels in moyamoya disease: a comparison of techniques. *European Radiology*.

[13] Sun W., Ruan Z., Dai X. (2018). Quantifying hemodynamic changes in moyamoya disease based on two-dimensional cine phase-contrast magnetic resonance imaging and computational fluid dynamics. *World Neurosurgery*.

[14] Zhang X., Su J., Gao C. (2018). Progression in vascular cognitive impairment: pathogenesis, neuroimaging evaluation, and treatment. *Cell Transplantation*.

[15] Tashiro R., Fujimura M., Kameyama M. (2019). Incidence and risk factors of the watershed shift phenomenon after superficial temporal artery-middle cerebral artery anastomosis for adult moyamoya disease. *Cerebrovascular Diseases*.

[16] Rennert R. C., Strickland B. A., Ravina K. (2019). Intraoperative assessment of cortical perfusion after intracranial-to-intracranial and extracranial-to-intracranial bypass for complex cerebral aneurysms using Flow 800. *Operative Neurosurgery*.

[17] Goertz L., Hof M., Timmer M. (2019). Application of intraoperative FLOW 800 indocyanine green videoangiography color-coded maps for microsurgical clipping of intracranial aneurysms. *World Neurosurgery*.

